# The Western Equine Encephalitis Lyophilized, Inactivated Vaccine: An Update on Safety and Immunogenicity

**DOI:** 10.3389/fimmu.2020.555464

**Published:** 2020-11-09

**Authors:** Maryam Keshtkar-Jahromi, Ronald B. Reisler, Jeannine M. Haller, Denise P. Clizbe, Robert G. Rivard, Anthony P. Cardile, Benjamin C. Pierson, Sarah Norris, David Saunders, Phillip R. Pittman

**Affiliations:** ^1^ Division of Infectious Diseases, Department of Medicine, Johns Hopkins University School of Medicine, Baltimore, MD, United States; ^2^ Division of Medicine, United States Army Medical Research Institute of Infectious Diseases, Fort Detrick, MD, United States

**Keywords:** vaccine, Western Equine Encephalitis, immunogenicity, clinical trial, inactivated

## Abstract

**Background:**

Western Equine Encephalitis (WEE) is a naturally acquired infection and potentially devastating bioweapon, with no specific human countermeasures. An experimental inactivated Western Equine Encephalitis Vaccine (WEEV; WEE TSI-GSD 210) has been used under an IND (investigational New Drug) protocol at the United States Army Medical Research Institute of Infectious Diseases (USAMRIID) since 1976.

**Methods:**

Over 24 years from 1987 to 2011, 876 subjects received 3 primary vaccine doses under 3 studies with 1,537 booster doses administered (FY87-8, phase 2, laboratory workers, vaccine lots 1-81-1, 1-81-2, and 2-1-91; FY99-12, phase 2 laboratory workers, lot 2-1-91; and FY09-02, phase 1 healthy volunteer, lot 3-1-92). Post-vaccination safety and immunogenicity [plaque reduction neutralization test 80% (PRNT_80_) > 1:40] were analyzed.

**Results:**

Overall PRNT_80_ response to the primary series in FY87-8 was 42% (326/770) but dropped to 16% (14/87) in FY99-12, prompting study FY09-02, which achieved 89% (17/19). The first booster response rate was 68% (814/1194) in FY87-8, 53% (171/324) in FY99-12, and 100% (10/10) in FY09-02. The majority of definitely related adverse reactions (AEs) were mild and local with no definitely related serious AEs. No laboratory acquired WEE infection was documented during this period despite 4 reported exposures in vaccinated subjects.

**Conclusion:**

The TSI-GSD 210 WEE vaccine was immunogenic, safe and well tolerated. Use of this vaccine could be considered in an emergency setting. Despite decades of safe and effective use under IND, full licensure is not planned due to manufacturing constraints, and a strategic decision to develop alternatives.

**Clinical Trial Registration:**

https://clinicaltrials.gov/, identifier NCT01159561.

## Introduction

Western Equine Encephalitis (WEE) virus is a mosquito-borne RNA virus in the *Alphavirus* genus of the Togaviridae family endemic to the Americas (1). First isolated in California in 1930 from the brain of a horse with encephalitis (2), WEE has previously caused significant morbidity and mortality in horses and humans (3). With the advent of a safe and effective multivalent veterinary vaccine, there’s been a dramatic decline in equine disease in the US (4, 5). Despite an effective IND (Investigational New Drug) vaccine candidate described herein, there are still no licensed human vaccines or therapeutics for WEE.

Human infection tends to be asymptomatic or mildly symptomatic with headache, nausea, vomiting, anorexia, and malaise. In some cases, central nervous system involvement (weakness, meningitis, and altered mental status) is apparent, with a very small percentage presenting with serious involvement including encephalitis, convulsion, confusion, coma, and subsequent death. The overall mortality rate is estimated at 3–7%, with severe neurologic sequelae expected in 15–30% of encephalitis survivors (2). The last confirmed human case occurred in Uruguay (South America) in 2009 (6) and in the US in 1994 (7). The virus has not been detected in mosquito surveillance in the US since 2008 (5). Nonetheless, WEE virus still remains a credible biothreat agent and can be aerosolized with potentially devastating neurologic morbidity and mortality (8). WEE virus is classified as a Category B bioterrorism agent by the US CDC.

Since the 1940s, the US Department of Defense has devoted a great deal of effort developing vaccines against alphaviruses [Venezuelan Equine Encephalitis (VEE), Eastern Equine Encephalitis (EEE), WEE, and Chikungunya] (9). For over 60 years, investigational alphavirus vaccines have been administered at the US Army Medical Research Institute of Infectious Diseases (USAMRIID) to at-risk laboratory workers *via* the Special Immunizations Program (SIP) (10). The SIP is the only program in the United States that provides investigational vaccines for laboratory workers exposed to hazardous pathogens (11). This program has provided a wealth of valuable information and a performance benchmark for comparing next generation vaccine products in development.

WEE vaccine (WEEV) was initially tested in 1970’s in controlled clinical trial studies at different dose schedules in volunteers at USAMRIID, and a published report demonstrated 88–92% adequate serological response to a two dose primary series (12). Based on these experiments, between 1976 and 1990, 359 laboratory workers were vaccinated at USAMRIID. The response rate was 50% as measured by PRNT80 ≥ 1:40 after primary series which increased to 60–70% after booster immunization (13). Since 1987, four lots (lots 1-81-1 and 1-81-2 manufactured in 1981, lot 2-1-91 manufactured in 1991, and lot 3-1-92 manufactured in 1992) have been administered within longitudinal, nonrandomized, non-stratified, observational trials at USAMRIID. In this report, data on safety and immunogenicity of WEEV from two phase 2 (FY87-8 and FY99-12) and one phase 1 study (FY09-02) spanning 1987–2011 are presented.

## Material and Methods

### Vaccine

The vaccine was derived from the attenuated CM-4884 strain of WEE virus. The product originated from supernatant fluids harvested from primary chicken embryo fibroblast cell cultures prepared from eggs infected with the attenuated CM-4884 strain. The supernatant fluid was harvested and filtered. The virus was inactivated with formalin, and residual formalin was neutralized by treatment with sodium bisulfate. Other components included neomycin sulfate and human serum albumin. The final product was lyophilized, sealed in vials under nitrogen, and stored at -20°C. Each 50 mL vial of vaccine was reconstituted with 21 mL of sterile water. The reconstituted product was stored at 2° to 8°C and used within 24 hours.

The test article was manufactured initially by the National Drug Company (NDC), a Division of Richardson Merrell Incorporated, and then by its successor, The Salk Institute - Government Services Division, Swiftwater, Pennsylvania. The latter product has been used at USAMRIID since 1970. All vaccine lots were manufactured by the same manufacturer using the same procedures. Each vaccine lot continued to pass all required potency tests based on GLP (Good Lab Practice) rodent vaccine studies conducted every 3 years and reviewed by FDA. All subjects received 0.5 mL of reconstituted vaccine subcutaneously in the upper outer aspect of the deltoid region.

To date, the vaccine has not been licensed by the United States Food and Drug Administration (FDA) but continues to be studied under an IND application (IND_2013).

### Study Subjects

Subjects enrolled in FY87-8 prior to 2000 were recruited at either USAMRIID or any of 34 extramural sites (remotely). From 2000 onward all subjects were enrolled at USAMRIID. Demographic data (age, gender, and race) were recorded. Inclusion and exclusion criteria through protocols FY99-12 and FY09-02 were similar. All three protocols limited the lower age to 18 years; FY99-12 and FY09-02 had an upper age limit of 55 years, whereas FY87-8 had no upper age limit. Pregnancy was ruled out by a negative urine pregnancy test on vaccination day. In the phase 1 FY09-02 study, subjects were excluded for previous WEE, EEE, VEE, and Chikungunya virus exposure (PRNT_80_ < 1:10). Subjects were required to comprehend and sign informed consent and to comply with all visits, testing, and adverse event reporting. Subjects with a history of allergies to formaldehyde, eggs, neomycin, streptomycin, human serum albumin, and sodium bisulfite were excluded. Also, excluded were those with history of blood product transfusion; receiving another vaccine or investigational product within 28 days of vaccination; and history of an immunodeficiency or taking immunosuppressive medications.

### Experimental Methods

**Figure 1:**
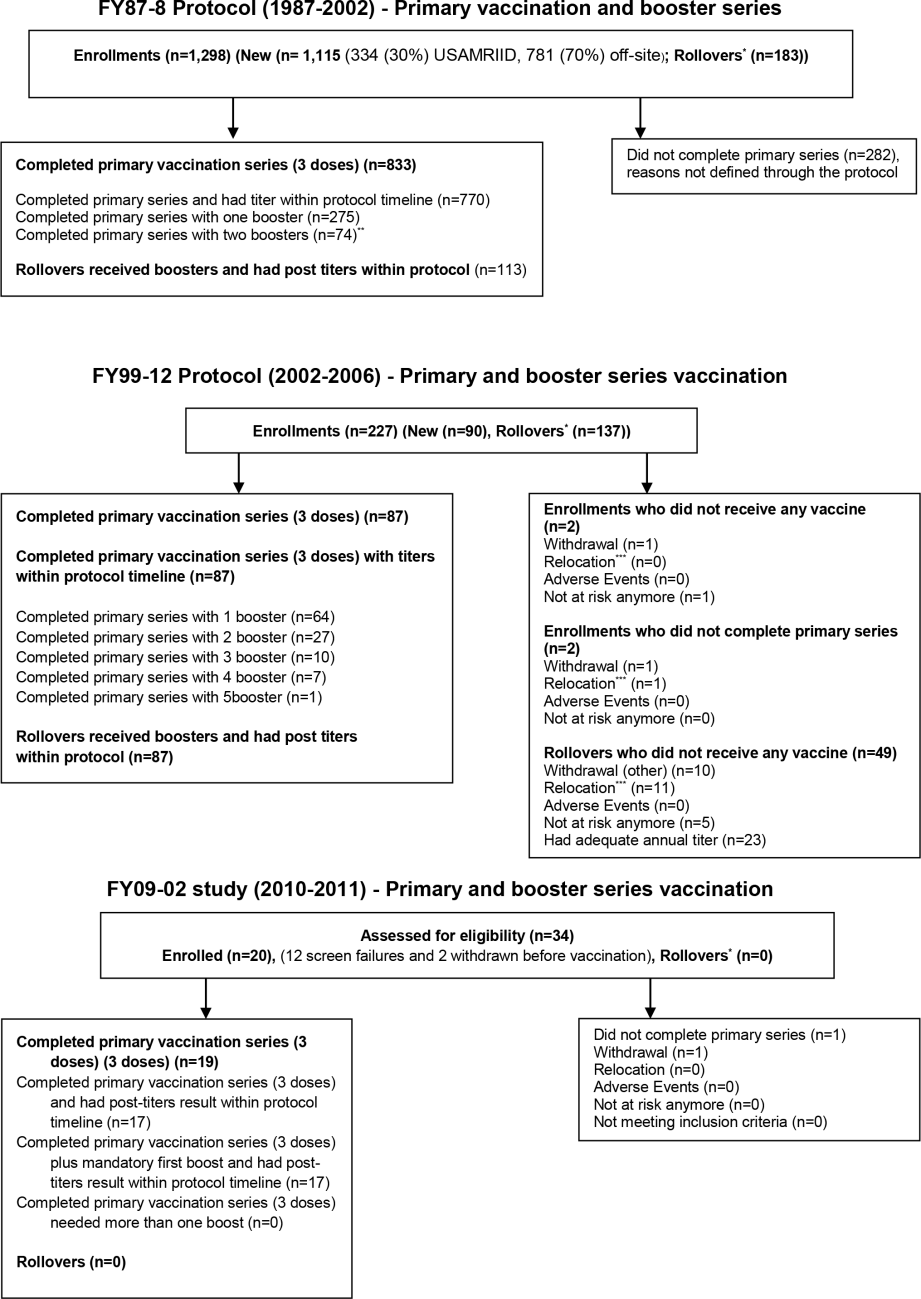
CONSORT diagram of study enrollment. ^*^Rollovers (subjects recruited from previous protocols); ^**^No more than two boosters required within this protocol; ^***^Relocation (subjects moved due to new job).

These studies were a continuation of previous WEE vaccine studies with the legacy WEE vaccine construct (**Figure 1**). They were, prospective, single-arm, open-label, nonrandomized, non-stratified trials of the safety and immunogenicity of inactivated WEE vaccine, TSI-GSD 210. Vaccination was performed based on volunteers’ stated need and risk of exposure to WEE. Vaccines were administered as an extra-layer of protection to normally used personal protective equipment within the Department of Defense Special Immunization Program (SIP). As a result, there was no sample size calculation for phase 2 employed in the trials, with volunteers enrolled sequentially until the protocols expired. Institutional Review Board (IRB) approval was obtained for all three protocols. Due to variations in protocol schedules and data collection tools over 24 years, methodology and data from each protocol are presented separately. All participants followed study protocols. Data for each study was collected from study final study reports independently. Institutional Review Board (IRB) approval was obtained for all three protocols.

#### Phase 2 Studies [FY87-8 (March 1987–February 2002) and FY99-12 (September 2002–August 2006)]

The primary vaccine series consisted of three doses (days 0, 7, and 28). Subjects with a post-primary or annual surveillance post-vaccination PRNT_80_ titer < 1:40 were offered a booster. Boosted subjects with recorded post-booster titer within 2 months were included in analysis. The FY87-8 utilized lots 1-81-1, 1-81-2, and 2-1-91 at USAMRIID and 34 off-site extramural locations. The three vaccine lots were sometimes interchangeably administered as boosters for the same subject. The switch from lots 1-81-1 and 1-81-2 to lot 2-1-91 was made primarily due to vaccine supply. Lot 1-81-1 was used earlier primarily at off-site locations and lot 2-1-91 later primarily at USAMRIID (p < 0.0001). The FY99-12 study was carried out using a single vaccine lot 2-1-91. Some subjects within FY99-12 study may have received their primary series under FY87-8 and rolled over into the FY99-12 protocol for annual titers.

#### Phase 1 Study [FY09-02 (February 2010–December 2011)]

The response rate to lot 2-1-91 decreased dramatically during study FY87-8 and particularly FY99-12, prompting a phase 1 trial in 2010 on a new lot from storage. The FY09-02 protocol utilized lot 3-1-92 to evaluate safety and immunogenicity of this new lot in healthy volunteers with naive immunity to all alphaviruses. The primary series were administered on days 0, 7, and 28 with a mandatory booster on day 180. PRNT_80_ titers were scheduled for days 56, 180, 210, and 360. A mandatory boost was added after average titers of <1:40 were observed following the primary series.

In the phase 1 study, the following statistical framework was used. Given sample sizes of 20, exact binomial 95% confidence intervals were calculated for proportions of prospective AE occurrence ranging from 5% (1/20 subjects) to 40% (8/20 subjects). Based on binomial approximation, and assuming a true prevalence of 10%, the probability of capturing an AE in at least one subject out of 20 was determined to be 87.8%. After lot 3-1-92 was proven to be safe and immunogenic, a phase 2 study (FY14-03) was initiated in 2015 which continues to accrue subjects, full result of this study will be published in a future report.

### Serology

Subjects’ sera were tested by plaque reduction neutralization test 80% (PRNT_80_) assays. This assay is considered the gold standard for detecting and measuring virus-specific neutralizing antibodies. The concentration of serum to reduce virus plaque formation by 80% compared to serum free viral suspension is calculated as the titer result. This assay was previously described by Mangiafico and Burke (14, 15) and modified for use with WEE virus. A conservative PRNT_80_ ≥1:40 was historically adopted as necessary for biocontainment suite entry to minimize risks. A “Responder” was defined as a PRNT_80_ ≥1:40 at any post-vaccination visit. Cellular immunity was not assessed in any of the protocols.

### Adverse Event

CTCAE (NCI Common Terminology Criteria for Adverse Events) was used to define AE and Serious AE (SAE). From 1987 to 2000, adverse advents (AEs) were passively collected at the time of follow-up. After 2000, AEs were actively solicited at four time points through day 28. In the FY99-12 and FY09-02 protocols, subjects were observed for 30-min post vaccination, and contacted *via* email, in person, or by phone on day 1 and weekly until day 28. The relationship between vaccine and each AE was determined by a physician (not related, unlikely, possible, probable, and definite).

### Safety Monitoring

Safety monitoring was conducted throughout the study; safety concerns were identified by continuous review of data by the PI (Principal Investigator), clinic staff, clinical monitor, and the research monitor. A Data Safety Monitoring Board was not required for these studies.

### Statistical Analysis

All data were collected from final study reports. Data were analyzed using SAS versions 8 and 9.1-9.4, depending on study and date of analysis. All hypothesis tests were at the 95% confidence level (two-tailed). The primary endpoint was the proportion of subjects who develop PRNT_80_ ≥ 1:40 at each scheduled time point over the entire study period. For all three studies, binomial proportions of subjects who developed PRNT_80_ ≥ 1:40 at each scheduled time point and exact 95% confidence intervals of these rates were calculated. Under the FY87-8 study, response rates were compared by multiple logistic regression adjusting for gender, age (<40 and ≥40), race (Caucasian and, non-Caucasian), lot (1-81-1, 1-81-2, and 2-1-91), and site (on-site vs. off-site administration of vaccine). Under the FY99-12 and FY09-02 study, response rates were compared by Fisher’s exact tests between gender, age (<40 and ≥40), and race (Caucasian and non-Caucasian) when applicable.

Geometric mean PRNT_80_ titers (GMT) and standard errors of the GMT at each scheduled time point were calculated for all three studies. Using log-transformed titers, any titers below the limit of detection were replaced with a value equal to 1.

For all three studies, rates of AEs were tabulated overall by type (local or systemic), severity, and relationship. Binomial proportions of these AEs and exact 95% confidence intervals were calculated.

#### Immunogenicity Analysis Population

Subjects who received all three primary or one boost dose and had a post-vaccination titer within 2 months of the last vaccine were included in analysis.

#### Safety Analysis Population

Only AEs that were determined to be definitely related to vaccine were included. All vaccinated subjects were included in safety analyses regardless of compliance with the protocol.

## Results

### Demographics

A total number of 4,920 vaccinations were administered to 1,362 subjects among the three protocols. Demographics of participants per protocol are presented in **Table 1**.

**Table 1:** Demographics of subjects receiving the WEE vaccine including description of the lots administered (1987–2011).

Protocol	FY87-8 (n = 1,115)	FY99-12 (n = 227)	FY09-02 (n = 20)
**Age range (years) (mean)**	19–74 (37)	20–73 (39)	22–54 (35)
<20	3 (<1%)	0	0
20–29	312 (28%)	57 (25%)	5 (25%)
30–39	375 (34%)	56 (25%)	8 (40%)
40–49	242 (22%)	70 (31%)	6 (30%)
50–59	121 (11%)	35 (15%)	1 (5%)
60–80	28 (2.5%)	9 (4%)	0
No data	34 (3%)	0	0
**Gender**			
Male	711 (64%)	151 (67%)	8 (42%)
Female	399 (36%)	76 (33%)	11 (58%)
No data	5 (<1%)		
**Race**			
Asian	31 (3%)	5 (2%)	0
Black	56 (5%)	13 (6%)	2 (10%)
Hispanic	25 (2%)	11 (5%)	1 (5%)
Native American or Pacific Islander	3 (<1%)	2 (<1%)	0
Caucasian	912 (82%)	192 (85%)	17 (85%)
Other	7 (<1%)	3 (<1%)	0
Unknown	81 (7.3%)	0	0
**Lots administered**			
1-81-1	1,751 (41%)	0	0
1-81-2	1,306 (31%)	0	0
2-1-91	1,196 (28%)	589	0
3-1-92	0	0	78
Total = 4,920	4,253	589	78

### Response to Primary Series

**Figure 2:**
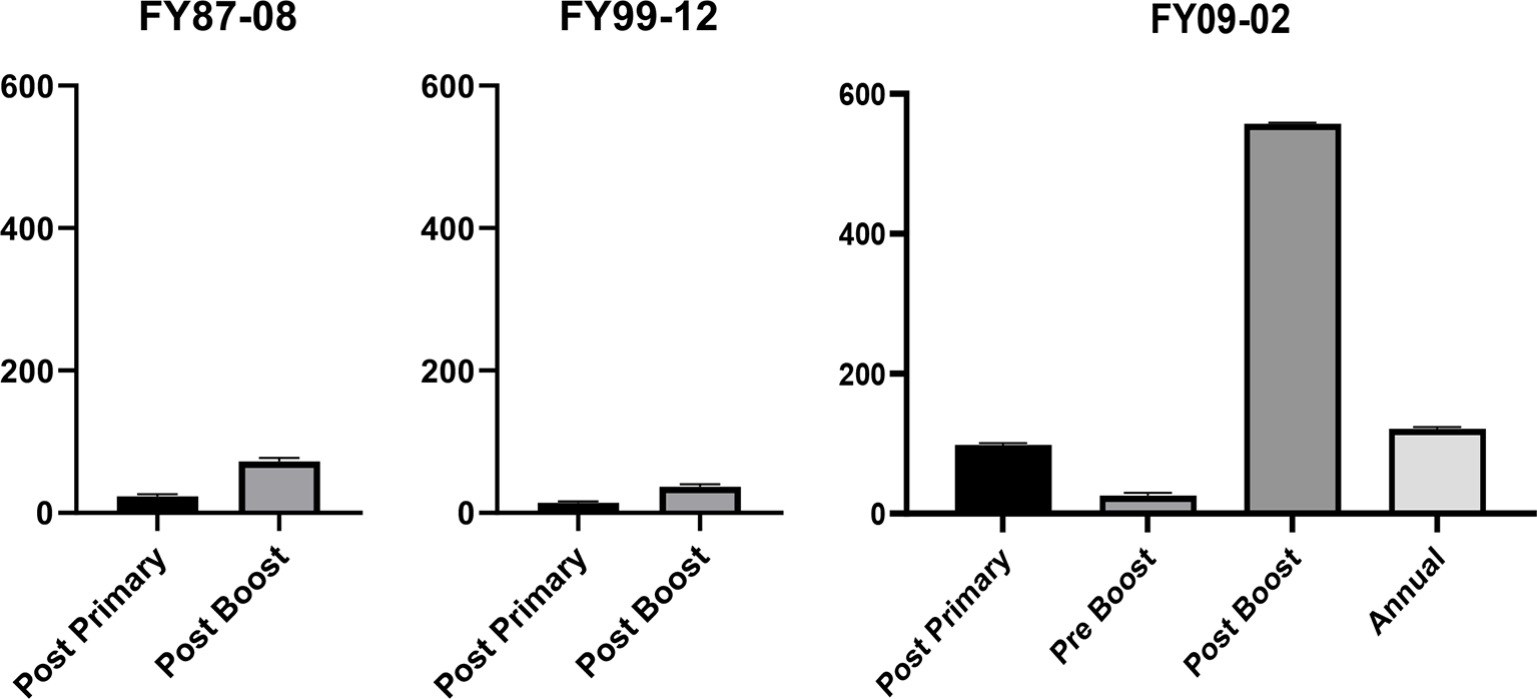
In FY87-08 protocol, post primary^*^ GMT (Geometric Mean Titers) was 23.3 [21.5, 25.2], and post booster^*^ GMT was 72.2 [63.3, 82.4]. In FY99-12, post primary^†^ GMT (Geometric Mean Titers) was 14.1 [12.1, 16.5], and post booster^†^ GMT was 36.9 [30.1, 45.3]. In the FY09-02 protocol, GMT day 56^‡^ was 98.1 [58.6, 164.3], day 180^‡^ (pre-6 month boost) was 25.8 [9.7, 68.9], day 210^‡^ (post 6-month boost) was 557.2 [376.8, 823.8], and day 360 was 121.3 [62.1, 236.8]. Thus, prior to the 6-month boost, the average titer was <1:40, justifying the need for the 6-month boost. ^*^Titers collected 14–56 days post vaccination. ^†^Titers collected 23–42 days post-vaccination. ^‡^Titers collected 21–35 days post-vaccination.

Overall, the PRNT_80_ response rate to the primary series was 42% (326/770) in FY87-8, 16% (14/87) in FY99-12, and 94% (16/17) in FY09-02 (**Table 2**). GMTs are shown for each of the 3 protocols (**Figure 2**).

**Table 2:** Overall response rates to WEEV following the 3^rd^ primary dose by subject demographics (1987–2011).

Protocol	FY87-8 [response rate (titer ≥ 1:40) (326/770 (42%)]	FY99-12 [response rate (titer ≥ 1:40) (14/87 (16%)]	FY09-02 [response rate (titer ≥ 1:40) (16/17 (94%)]
**Gender**			
Male	187/491 (38%)	6/55 (11%)	7/7 (100%)
Female	135/274 (49%)	8/32 (25%)	9/10 (90%)
Unknown	4/5 (80%)	0	0
P-value	**p = 0.0215**	p = 0.1334	p = 1.0
**Age (Years)**			
<40	217/528 (41%)	9/60 (15%)	11/11 (100%)
≥40 (23 unknown not included in p-value comparison)	91/219 (42%)	5/27 (18.5%)	5/6 (83%)
P-value	p = 0.9607	p = 0.7539	p = 0.3529
**Race** (46 Unknown not included in p-value comparison)			
Non-caucasians	24/88 (27.2%)	3/22 (13.6%)	1/2 (50%)
Caucasians	269/636 (42.3%)	11/65 (16.9)	15/15 (100%)
P-value	**p = 0.0119**	p = 1.000	NA

In FY87-8, response rates were compared by logistic regression. In FY99-12 and FY09-02, response rates were compared by Fisher’s exact tests. Statistically significant p-values are in bold.

### Response Rate to Boosters

The overall booster response rate across all analyzable doses was 68% (814/1194) in FY87-8, 53% (171/324) in FY99-12 and 100% (10/10) in FY09-02 (**Table 3**).

**Table 3:** Overall response rates to WEEV booster doses by subject demographics (1987–2011).

Protocol	FY87-8 [overall response rate (814/1194 (68%)] (titer ≥ 1:40)	FY99-12 [overall response rate (171/324 (53%)] (titer ≥ 1:40)	FY09-02 [response rate (10/10 (100%)] (titer ≥ 1:40) (after 6-month booster)
**Gender**			
Male	556/836 (66.5%)	117/235 (50%)	5/5 (100%)
Female	257/357 (72%)	54/89 (60.7%)	5/5 (100%)
P-value	p = 0.2516	p = 0.0827	NA
**Age (years)**			
< 40	489/703 (69.6%)	94/174 (45%)	6/6 (100%)
≥ 40	323/488 (66.2%)	77/150 (57.3%)	4/4 (100%)
P-value	p = 0.0717	p = 0.6563	NA
**Race**			
Non-Caucasians	84/124 (68%)	29/43 (67.4%)	1/1 (100%)
Caucasians	714/1047 (68%)	142/281 (50.5%)	9/9
P-value	p = 0.7096	**p = 0.0485**	NA

FY87-8 response rates were compared by logistic regression. FY99-12 and FY09-02 response rates were compared by Fisher’s exact tests.Statistically significant p-values are in bold.

#### Initial Non-Responders (after Three Dose Primary Series)

In protocol FY87-8, there were 333 (43%) non-responders. Response rate to the first and second booster were 63% (174/275) and 45% (33/74), respectively. In protocol FY99-12, there were 74 (84%) non-responders. Response rates to first, second, third, fourth, and fifth boosters were 45% (29/64), 52% (14/27), 20% (2/10), 0% (0/7), and 100% (1/1), respectively. In protocol FY09-02, 1 of 17 subjects was classified as a non-responder, and the response rate to the first boost was 100% (1/1).

### Effects of Gender, Age, Race, On-Site Study Participation (FY87-8), and Vaccine Lot (FY87-8)

Females had a significantly higher primary series response during FY87-8. Primary series response rate was significantly higher in Caucasians in FY87-8, but booster response rates were significantly higher in non-Caucasians in FY99-12. Different age groups responded to vaccine similarly. Volunteers vaccinated outside USAMRIID had a higher response rate after primary series within FY87-8 (p = 0.0326) (**Table 4**). Lot 2-1-91 had a significantly lower response rate than either Lot 1-81-1 or Lot 1-81-2, while there was no significant difference between previously manufactured lots (1-81-1 and Lot 1-81-2) in the FY87-8 protocol (**Table 4**).

**Table 4:** Differences in response rates after third primary dose and boosters in the FY87-8 study protocol by lot and study site.

Lots, Site	Response rate (titer ≥ 1:40) [326/770 (42%)] after primary series	Response Rate [814/1194 (68%)] (titer ≥ 1:40) after booster
**Vaccine Lot**		
1-81-1	185/347 (53%)	300/388 (77.3%)
2-1-91	39/168 (23%)	287/496 (58%)
P-Value	p = 0.0003	p <.0001
1-81-2	102/255 (40%)	227/310 (73%)
2-1-91	39/168 (23%)	287/496 (58%)
P-Value	p = 0.0114	p <.0001
1-81-1	185/347 (53%)	300/388 (77.3%)
1-81-2	102/255 (40%)	227/310 (73%)
P-value	p = 0.0867	p = 0.6481
**Study Site**		
USAMRIID	67/252 (26.6%)	418/666 (62.8%)
Off-site	259/518 (50%)	396/528 (75%)
P-value	p = 0.0326	p = 0.1519

Response rates were compared by logistic regression.

### Safety Data

Summaries of all definitely related AEs in the three protocols are presented in **Tables 5**, **6**.

**Table 5:** Local Adverse Events (AEs) definitely related to the vaccine (1987–2011).

Local Adverse Events	FY87-8	FY99-12	FY09-02	Total (%)	Overall
Site erythema	70	10	26	106 (36%)	27%
Site pruritus	25	10	10	45 (15%)	12%
Site tenderness	24	8	11	43 (15%)	11%
Site induration	26	4	2	32 (11%)	8%
Site warmness/inflammation	23	2	1	26 (9%)	7%
Site edema/swelling	10	3	7	20 (7%0	5%
Site bruise	5	3	6	14 (5%)	4%
Stinging	1	1	0	2 (1%)	<1%
Numbness	2	0	0	2 (1%)	<1%
Site tingling	0	1	0	1 (<1%)	<1%
Knot at injection site	1	0	0	1 (<1%)	<1%
Site papule	1	0	0	1 (<1%)	<1%
Site lesion	1	0	0	1 (<1%)	<1%
Total	189	42	63	294 (100)	76%

**Table 6:** Systemic Adverse Events (AEs) definitely related to the vaccine (1987–2011).

Systemic adverse events	FY87-8	FY99-12	FY09-02	Total	Overall
Headache	15	1	0	16 (17%)	4%
Malaise	8	0	0	8 (9%)	2%
Fatigue	8	0	0	8 (9%)	2%
Fever	7	0	0	7 (8%)	2%
Myalgia	7	0	0	7 (8%)	2%
Sore throat	5	0	0	5 (6%)	1%
Hives	5	0	0	5 (6%)	1%
Flushed	4	0	0	4 (4%)	1%
Nausea	4	0	0	4 (4%)	1%
Rash	4	0	0	4 (4%)	1%
Lymphadenopathy	2	1	0	3 (3%)	<1%
Chalky taste	0	2	0	2 (2%)	<1%
Anorexia	2	0	0	2 (2%)	<1%
Diarrhea	2	0	0	2 (2%)	<1%
Neck stiffness	2	0	0	2 (2%)	<1%
Sore neck	2	0	0	2 (2%)	<1%
Polydipsia	0	0	1	1 (1%)	<1%
Tongue sores	0	1	0	1 (1%)	<1%
Eyelids swollen	1	0	0	1 (1%)	<1%
General swelling	1	0	0	1 (1%)	<1%
Back pain	1	0	0	1 (1%)	<1%
Arthralgia	1	0	0	1 (1%)	<1%
Dizziness	1	0	0	1 (1%)	<1%
Lethargy	1	0	0	1 (1%)	<1%
Flu-like symptoms	1	0	0	1 (1%0	<1%
Chills	1	0	0	1 (1%)	<1%
Night sweats	1	0	0	1 (1%)	<1%
Total	86	5	1	92 (100%)	24%

#### FY87-8

A total of 4,253 vaccine doses were administered to 1,273 subjects. Subjects received multiple doses based on need. Of the 4,253 doses administered, 4,059 doses (1,510 on-site and 2,549 off-site) did not result in reported AEs. The remaining 194 doses in 144 subjects had at least one AE for a total of 566 AEs. Overall, 49 out of 130 vaccinated subjects (37.7%) at USAMRIID reported at least one AE (either local or systemic). The majority of reactions occurred on the day of or within the first 3 days following vaccination. Of the 566 AEs, 275 (49%) were definitely related to the vaccine. Of the definitely related AEs, 189 (69%) were local (**Table 5**), and 86 (31%) were systemic (**Table 6**). Only eight subjects had related AEs on multiple injections, some of which were not consecutive. Only one subject developed vaccine-related AEs after three consecutive administrations. One other subject developed vaccine-related AEs after 4 consecutive vaccinations. There was a pattern of increasing severity in related AEs in only one subject. No subject experienced an SAE, though five subjects experienced AEs related to WEE vaccine graded as severe (headache, arthralgia, swelling, and erythema).

#### FY99-12

A total of 589 vaccine doses were administered to 176 subjects. Subjects received multiple doses of vaccine based on need. Of the 176 vaccinated subjects, 37 subjects (21%) had at least one AE. A total of 221 AEs were reported. Females reported more AEs than males (p = 0.0448). Of the 221 AEs, 47 (22%) in 10 subjects were assessed as definitely related to vaccination. Of the 47 definitely related AEs, 42 (89%) were local (**Table 5**), and 5 (11%) were systemic (**Table 6**). No related SAEs or severe AEs were reported. Only one subject developed vaccine related AEs after three consecutive vaccinations. No subject developed vaccine related AEs after four or more consecutive vaccinations they received. There was no clear pattern of increasing or decreasing severity in related AEs with subsequent vaccinations in this subject.

#### FY09-02

A total of 78 doses of vaccine were administered to 20 subjects during the phase 1 study. All subjects (100%) developed at least one related AE (either systemic or local) with a total of 163 reported AEs. All subjects received 4 vaccinations and all subjects (100%) developed at least one AE (either systemic or local) with total of 163 reporting AEs. Six subjects reported related AEs after all vaccine doses. Only three subjects had moderate severity AEs. For all three subjects, none of the moderate AEs were the same as those they had previously experienced as mild. There no clear changes in AE severity with subsequent vaccinations in these subjects. Of the 163 AEs, 64 (39%) were definitely related to the vaccine. Of the 64 definitely related AEs, 63 (98%) were local (**Table 5**), and 1 (2%) was systemic (**Table 6**). There were no related SAEs or severe AEs reported, and no statistically significant differences between the percentages of women and men reporting specific AEs.

## Discussion

Currently, there is no FDA approved preventive or therapeutic medical countermeasure against WEE virus. The TSI-GSD 210 vaccine, IND 2013, remains the only human vaccine developed and used in clinical trials since 1970 at USAMRIID (12). Over 50 years, challenges with the vaccine included declining response rates among aging lots and determining the optimal vaccination strategy. Nonetheless, this legacy vaccine product proved resilient, generating what are thought to be protective levels of immunity among a large cohort of healthy laboratory worker volunteers.

In this report, we presented data from three most recent (FY87-8, FY99-12, and FY09-02) protocols. In 1970, two dose primary WEE vaccine series were administered to 18 healthy volunteers with two different dose schedules, and 88% to 92% achieved adequate immunity based on ≥1.7 LNI (mean log10 serum neutralizing indices) (12). From 1987 to 2011, primary series response rates (PRNT_80_ ≥ 1:40) ranged from 16–89% (1987–2011). Were we to use lower standards for PRNT comparable to those for similar indications (PRNT_50_ ≥ 1:40, or PRNT_50_ ≥ 1:10, or PRNT_80_ ≥ 1:10), rates of adequate immunogenic response would be higher. While there were a number of individuals who maintained adequate titers for a prolonged period of time, many individuals required frequent boosters to maintain a PRNT_80_ ≥ 1:40. This vaccine still continues to be administered under FY14-03 (open protocol using lot 3-1-92) providing acceptable immunogenicity [PRNT_80_ ≥ 1:40 response rate of 93% (13/14) to primary series and 85% (11/13) to boosters (unpublished data)].

**Figure 3:**
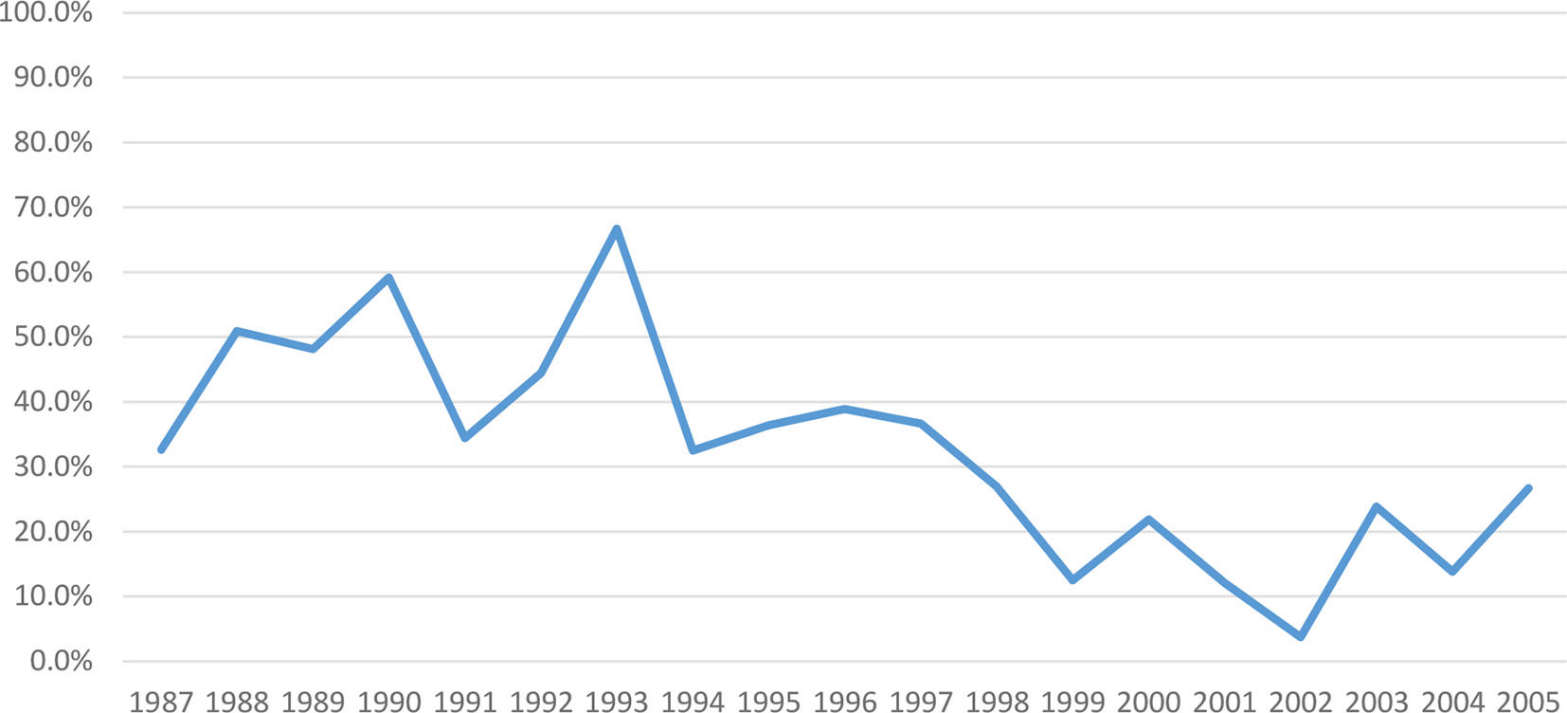
Post-Primary series PRNT_80_ immune response to WEE vaccine by year within FY87-8 and FY99-12 (1987–2005). The immunogenicity of WEE TSI-GSD 210 appeared to decline from 53 to 23%.

**Figure 4:**
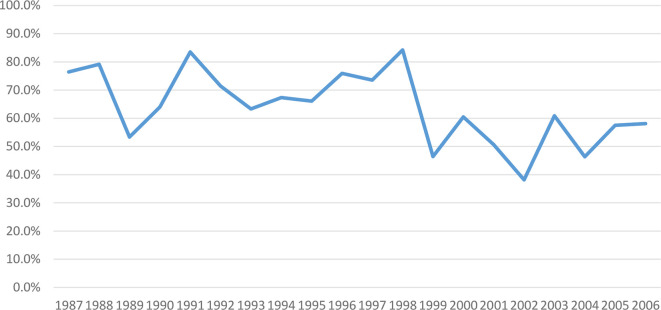
Post-boosters PRNT_80_ immune response to WEE vaccine by year within FY87-8 and FY99-12 (1987–2006). Immunogenicity of WEE TSI-GSD 210 appeared to decline from 77–58%.

During FY87-8, investigational product was switched to lot 2-1-91 in 1993 due to exhaustion of vaccine older lot supply. We subsequently observed declining response rates to the primary series from 53 to 23% and to boosters from 77 to 58% (1987–2006) (**Figures 3**, **4**), though all lots passed GLP rodent potency testing requirements. Lot 2-1-91 was manufactured 10 years after previously used lots by the same manufacturer and with the same contents. Lower response rates could be due to different fixation processes leading to different particle sizes, or simply due to rapid lot degradation. Thus, a different lot (3-1-92) was used, requiring evaluation in a phase 1 trial (NCT01159561) (2010-2011), and showed improved immunogenicity. Subsequently, a new phase 2 study with this new lot has been enrolling since December 2015 (NCT02466750). These efforts over many years have enabled SIP to keep offering this vaccine to at-risk laboratory workers as next-generation vaccines are developed.

Multiple logistic regression analysis of WEE immune response rates to primary series and boosters yielded non-significant differences by age. Caucasian, female and outside USAMRIID subjects were more likely to have adequate response to primary series vaccinations in protocol FY87-8 only.

We have also observed this vaccine to be safe, with most of definitely related reactions being categorized as mild, local. The majority of systemic reactions were categorized as mild, and no SAEs related to WEEV were reported. Safety data was collected passively in FY87-8, the protocol with the largest number of subjects and longest duration. Despite increasing rates of total AEs from 13 to 37% after changes in AE collection from passive to active in 1999 (FY99-12), rates of definitely related AEs remained stable (6% to 7%). Overall, rates of AEs were comparable to commonly used inactivated viral vaccines such as inactivated influenza vaccine [local (76 vs. 64%); systemic (24 vs. 31%) per vaccination] (16). No consistent pattern of changes in the number or severity of related AEs was observed.

Historically, at least two fatal (in the 1930’s) and one non-fatal laboratory acquired WEE infections have been reported in the literature (17–19). USAMRIID has never had a documented case of occupationally acquired WEE infection. Four vaccinated individuals reported potential occupational exposures to WEE virus, but none developed signs of disease (20). While this suggests modest supportive evidence for vaccine effectiveness, the high level of institutional biosafety practice standards minimizes the risk of actual exposure.

At the time of writing, there is no longer an identified manufacturer for this vaccine. Only a limited stock of the vaccine remains, with no current capability to produce additional product. This decision was based in part on USAMRIID’s prior experience with immune interference following sequential alphavirus vaccine inoculations (21). The current strategy is to develop next-generation vaccines such as the novel recombinant and experimental trivalent alphavirus vaccine (EEE, VEE, and WEE) currently in development (*Clinicaltrials.gov* NCT03879603 and NCT04131595). While several other human vaccine candidates are currently in development in non-human primates, no human trials of next generation candidates have yet been published (22, 23).

In summary, WEEV has proven safe and immunogenic for over 50 years, even many years after manufacture and storage under appropriate conditions. It is appropriate as an adjunct to rigorous biosafety practices to prevent infection in at-risk laboratory personnel. Ongoing use of this vaccine is justified pending development of a clinically viable alternative. Use of the vaccine could be considered in an emergency setting.

## Data Availability Statement

The datasets presented in this article are not readily available because: The data is property of US government and requires special permission to be shared. Requests to access the datasets should be directed to sarah.l.norris2.civ@mail.mil.

## Ethics Statement

The studies involving human participants were reviewed and approved by USAMRIID Human Research Protection Office, Office of Research Protections Headquarters, U.S. Army Medical Research and Development Command. The patients/participants provided their written informed consent to participate in this study.

## Author Contributions

MK, RR, RGR, and PRP contributed to conception and design of the study. SN, DC, BP, and JH organized the database. SN performed the statistical analysis. MK and RR wrote the first draft of the manuscript. MK, DS, AC, and MK wrote sections of the manuscript. PRP was Assoc Invest and PI on Protocol FY87-8 and wrote Protocol FY99-12. All authors contributed to the article and approved the submitted version.

## Funding

This work was supported by the US Army Medical Research and Development Command (USAMRDC); Defense Threat Reduction Agency (DTRA); multiple extramural partners with at-risk laboratory personnel; and USAMRIID. Funding for safety, potency testing, and product storage was provided to USAMRIID by the Joint Product Executive Office for Chemical, Biological, Radiological, and Nuclear Defense (JPEO-CBRND).

## Disclaimer

Opinions, interpretations, conclusions, and recommendations are those of the authors and are not necessarily endorsed by the U.S. Army.

## Conflict of Interest

The authors declare that the research was conducted in the absence of any commercial or financial relationships that could be construed as a potential conflict of interest.
